# Gradenigo syndrome in a child: case report and literature review

**DOI:** 10.1097/RC9.0000000000000371

**Published:** 2026-03-12

**Authors:** Badr Saout Arrih, Walid Bijou, Myriam Loudghiri, Sami Rouadi, Redallah Abada, Mohamed Mahtar

**Affiliations:** Department of Otolaryngology, Head and Neck Surgery, Faculty of Medicine and Pharmacy, Ibn Rochd University Hospital, Hassan II University, Casablanca, Morocco

**Keywords:** abducens nerve, child, Gradenigo syndrome, mastoidectomy, otitis media, petrous apex, trigeminal nerve

## Abstract

**Introduction and Importance:**

Gradenigo syndrome is a rare but potentially serious complication of suppurative otitis media, characterized by the classic triad of suppurative otorrhea, abducens nerve (VI) paralysis, and pain in the trigeminal nerve (V) territory. Although the advent of antibiotic therapy has significantly reduced its incidence, this condition remains relevant, particularly in children, requiring early recognition and appropriate multidisciplinary management.

**Case Presentation:**

We report the case of a 13-year-old girl who presented with a painful swelling behind her right ear that had been developing for a year, accompanied by headaches, pain behind her right eye, and earache, along with a fever. Clinical examination revealed severe bulging of the right tympanic membrane, inflammatory retro auricular swelling, and decreased right ocular mobility in abduction with diplopia, suggestive of abducens nerve involvement. Imaging (CT scan of the temporal bone and brain) confirmed the diagnosis, showing a right mastoid subperiosteal abscess with complete opacification of the tympanic cavity, bone lysis of the external wall of the mastoid, and filling of the petrous apex. Treatment consisted of intravenous antibiotic therapy (third-generation cephalosporin (Ceftriaxone 50 mg/kg/day IV) and metronidazole (30 mg/kg/day IV)) for 15 days, short-term corticosteroid therapy, drainage of the collection, and drainage mastoidectomy due to persistent symptoms.

**Clinical Discussion:**

This case perfectly illustrates the contemporary evolution of Gradenigo syndrome, where incomplete forms predominate. The pathophysiology involves the spread of infection from the middle ear to the petrous apex. Current microbiological data identify Fusobacterium necrophorum as the most common pathogen, justifying the systematic use of an anti-anaerobic agent. Modern management combines prolonged antibiotic therapy with surgery. Our patient’s progress, marked by the disappearance of retro-orbital pain on the 5th postoperative day and complete recovery of ocular mobility on the 10th day, is consistent with the usual recovery times (median of 11 days after surgery).

**Conclusion:**

Gradenigo syndrome remains a serious complication of otitis media requiring early diagnosis and multidisciplinary management. The evolution of clinical presentations toward incomplete forms requires an adaptation of traditional diagnostic criteria. With appropriate management combining prolonged antibiotic therapy and surgery when necessary, the prognosis is generally favorable. This case highlights the importance of maintaining a high degree of clinical suspicion in the presence of any complicated otitis in children, particularly when associated neurological signs are present.

## Introduction

Gradenigo syndrome, first described in 1904 by Giuseppe Gradenigo^[^1^]^, is a rare but potentially serious complication of suppurative otitis media. This nosological entity is characterized by the classic triad of suppurative otorrhea, abducens nerve (VI) paralysis, and pain in the trigeminal nerve (V) distribution area, resulting from the spread of infection to the petrous apex of the temporal bone^[^2^]^.

The advent of antibiotic therapy has significantly altered the epidemiology of this condition, transforming what was once a fatal disease into an exceptional complication^[^3^]^. However, contemporary data show that this complication remains relevant, particularly in pediatric populations^[^4,5^]^.

The petrous apex, which is pneumatized in approximately 30% of the population^[^6^]^, has close anatomical connections with the Gasser ganglion of the trigeminal nerve and the abducens nerve passing through Dorello’s canal^[^7^]^. This proximity explains the characteristic neurological symptoms associated with inflammatory spread, a process that can progress to osteomyelitis and intracranial complications^[^8,9^]^.

Contemporary clinical presentations often deviate from the classic description, with incomplete forms predominating and requiring a high degree of diagnostic suspicion^[^10,11^]^. Modern imaging, particularly computed tomography (CT) and magnetic resonance imaging (MRI), now allows for early detection of signs of apical petrositis^[^10^]^.

The aim of this publication is to report the case of a 13-year-old adolescent girl presenting with Gradenigo syndrome secondary to complicated otomastoiditis, discussing the diagnostic and therapeutic aspects of this rare complication in light of the current literature.

This case report has been reported in line with the Surgical CAse Report (SCARE) checklist. ^[12]^

## Case report

We present the case of a 13-year-old girl with a history of otitis media, no other medical conditions, and no specific medication use. She presented to our Ear, Nose, and Throat (ENT) department with a painful swelling behind her right ear.

Her medical history dates back 1 year, when she began experiencing headaches, pain behind her right eye, and earache in her right ear, accompanied by fever and a good general condition. On general physical examination, the patient was conscious, with good cardiovascular and respiratory function (Table 1).

**Table 1:** Timeline of clinical events.

Time point	Symptoms/clinical findings	Investigations	Interventions	Outcomes
1 year prior	Headaches, retro-orbital pain (right), right earache, fever	None	None documented	Symptoms persisted
Initial presentation (Day 0)	Painful swelling behind right ear, persistent symptoms	Otoscopy: severe bulging of right tympanic membrane Ophthalmological exam: decreased right eye abduction, diplopia	None	Diagnosis suspected
Day 0	Constitutional symptoms present	CT temporal bone: subperiosteal abscess (12 mm thick, 56 × 60 mm), complete tympanic cavity opacification, mastoid wall lysis, petrous apex involvement Brain CT: confirmed findings	Admission IV antibiotics initiated (Ceftriaxone 50 mg/kg/day + Metronidazole 30 mg/kg/day) Corticosteroids (5 days) Drainage of periosteal collection	Cultures negative
Day 15	Persistent symptoms despite medical therapy	Clinical reassessment	Mastoidectomy performed	Surgical drainage successful
Postoperative Day 5	Retro-orbital pain resolved	Clinical monitoring	Continued oral antibiotics (15 days)	Pain-free
Postoperative Day 10	Normal ocular mobility restored	Ophthalmological reassessment	Antibiotics continued	Complete neurological recovery
Follow-up	Complete resolution of all symptoms	Clinical evaluation	Treatment completed	Excellent outcome

**Figure 1.**
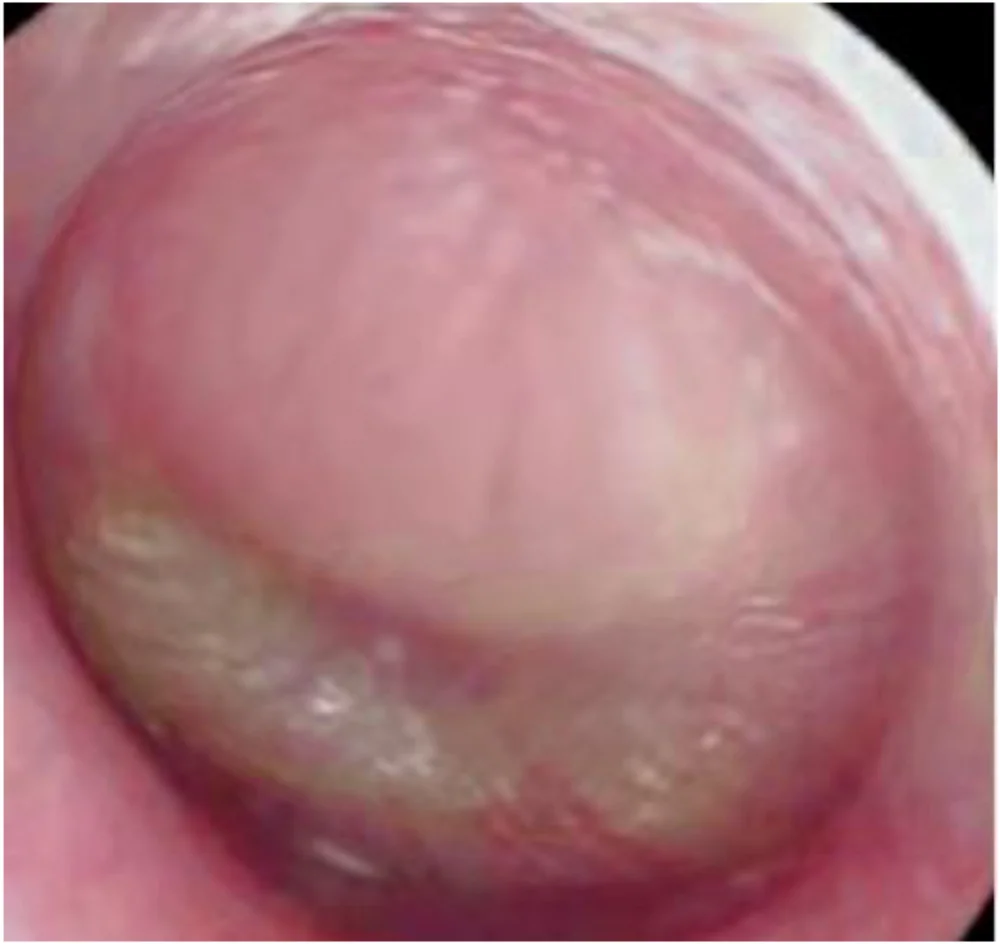
Otoscopic view of the right ear demonstrating severe bulging of the tympanic membrane. The membrane appears tense and hyperemic, indicating middle ear effusion under pressure. This finding is characteristic of acute otitis media and represents the initial stage of the infectious process that subsequently spread to the petrous apex.

**Figure 2.**
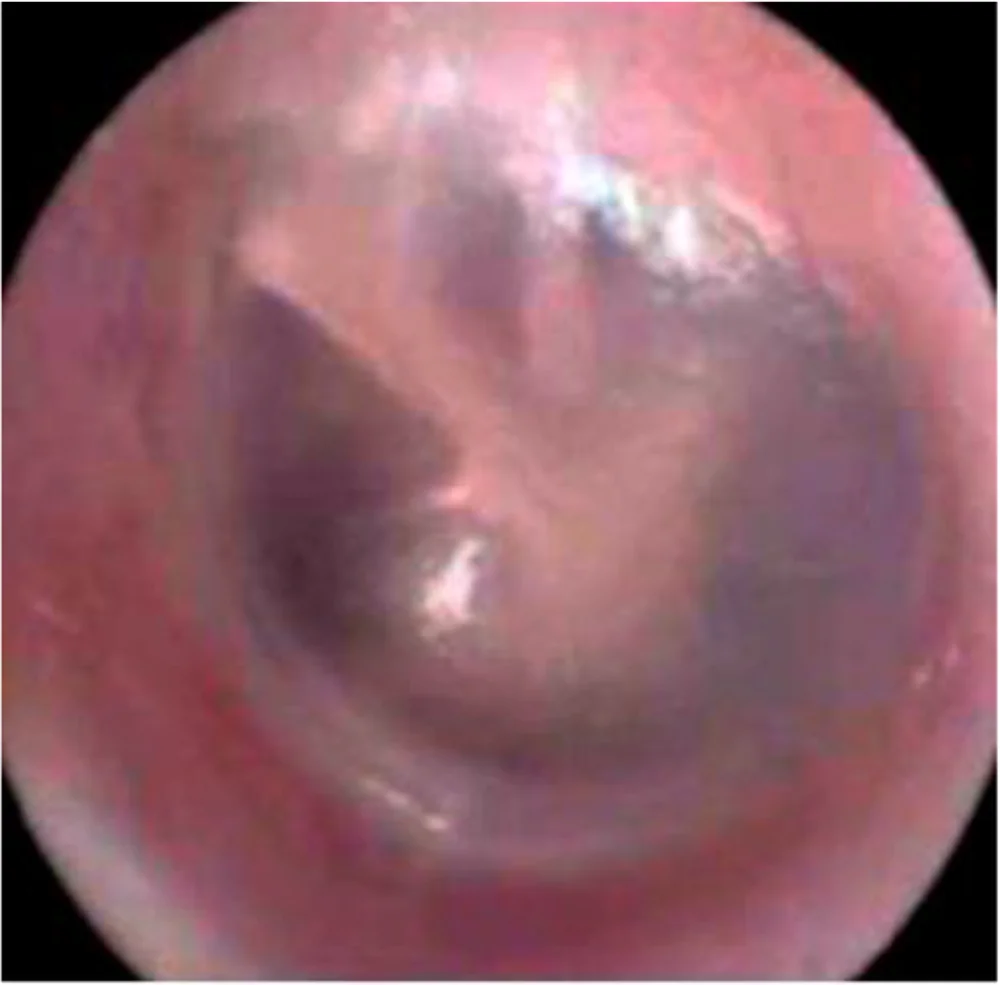
Normal otoscopic appearance of the left ear for comparison. The tympanic membrane shows normal translucency, appropriate light reflex, and clear visualization of the malleus handle and umbo, confirming that the infectious process was unilateral.

During the ENT physical examination, otoscopy of the right ear revealed severe bulging of the right tympanic membrane, consistent with middle ear effusion (Fig. 1). Otoscopy of the left ear was normal (Fig. 2). There is an inflammatory swelling behind the right ear without effacement of the retroauricular line. Rhinoscopy reveals large adenoids filling the cavum, which appears normal. Endobuccal examination reveals nasopharyngitis. The cervical examination is normal, with no palpable lymphadenopathy (Table 1).

**Figure 3.**
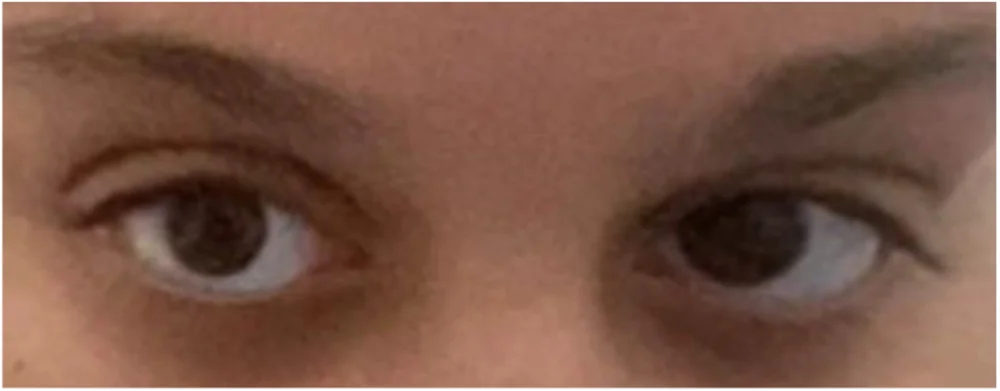
Clinical photograph demonstrating reduced right ocular mobility in abduction, indicating abducens nerve (cranial nerve VI) palsy. Note the inability of the right eye to move laterally beyond the midline, resulting in horizontal diplopia. This finding, combined with retro-orbital pain and otorrhea, constitutes Gradenigo syndrome.

Ophthalmological examination reveals decreased right eye mobility in abduction with right diplopia (Fig. 3).

The prolonged duration of symptoms (1 year) initially suggested a chronic process, making the acute neurological deterioration unexpected and challenging to diagnose. The incomplete presentation without active otorrhea further complicated the diagnostic process (Table 1).

**Figure 4.**
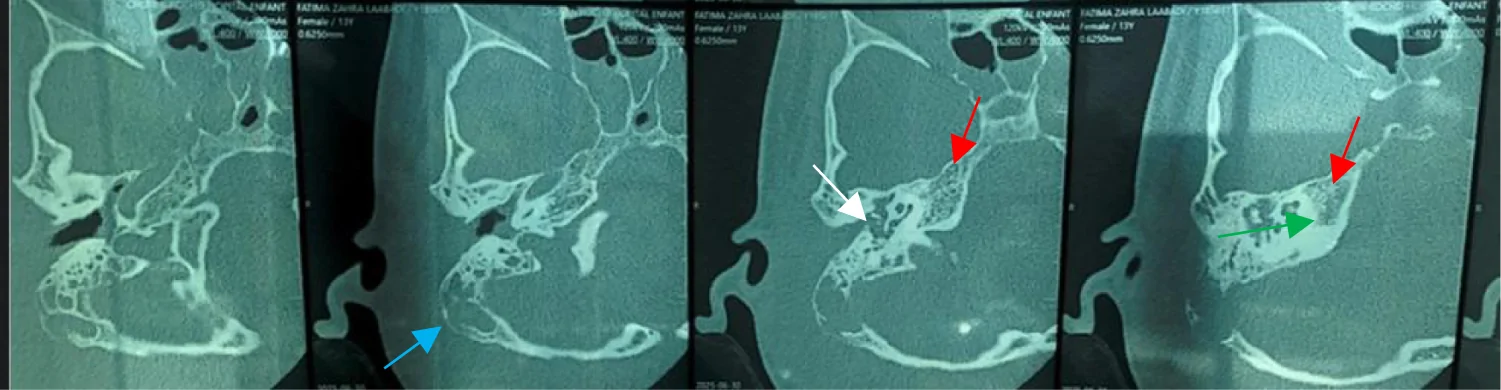
Axial CT scan of the right temporal bone with contrast enhancement showing: (white arrow) complete opacification of the tympanic cavity with fluid/inflammatory material; (blue arrow) bone lysis of the external wall of the mastoid indicating osteitis; (green arrow) intact ossicular chain without erosion.

Note: Green arrow has been corrected to properly point to the ossicles; (red arrow) inflammatory opacification of the petrous apex, the pathognomonic finding in Gradenigo syndrome.

Given this clinical picture, a CT scan of the right temporal bone was requested, showing a subperiosteal collection opposite the mastoid, with a thick wall enhanced after contrast injection, and hypodense content with multiple partitions. It measured 12 mm at its thickest point and extended 56 mm in the axial plane and 60 mm in the coronal plane. There was also complete opacification of the tympanic cavity and all the mastoid cells, associated with bone lysis of the external wall of the mastoid was observed, with preservation of the ossicular chain. There was also filling of the apex of the petrous bone (Fig. 4) (Table 1).

HIGHLIGHTS

- Gradenigo syndrome is a rare complication of otitis media, with only 22% of pediatric cases presenting with the complete classic triad at initial diagnosis.
- Modern imaging (CT and MRI) allows early detection of apical petrositis, revealing obliteration of the petrous apex in 49% of cases and an inflammatory process in 84% of cases.
- Fusobacterium necrophorum is emerging as the most common pathogen (22% of isolates), justifying the systematic addition of anti-anaerobic treatment to the antibiotic protocol.
- Multidisciplinary management combining prolonged antibiotic therapy (median of 21 days IV) and surgery (mastoidectomy in 72% of cases) leads to clinical improvement in 98% of cases.
- The prognosis is excellent, with complete neurological recovery being the norm, in contrast to the historical mortality rate of 50% in the pre-antibiotic era, which has now been reduced to 2%.

**Figure 5.**
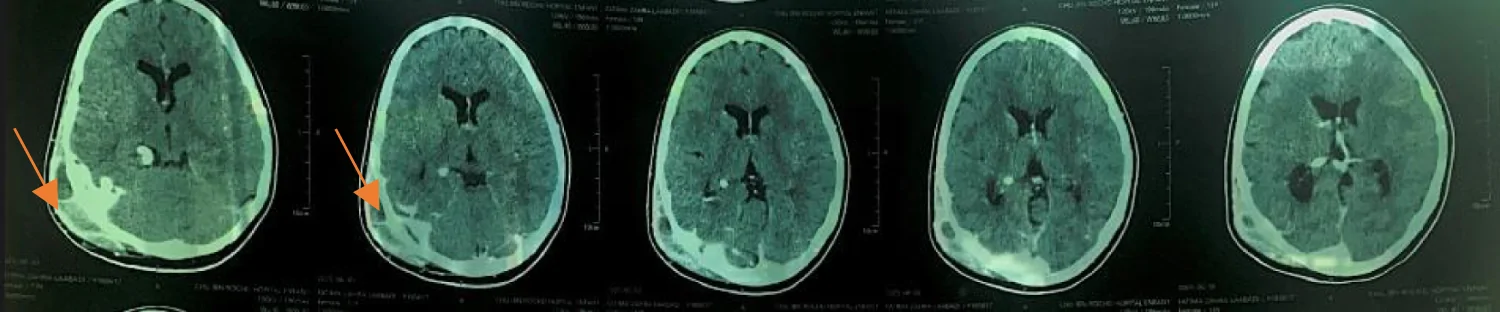
Axial CT scan of the brain at soft tissue windows demonstrating (orange arrow) the subperiosteal mastoid abscess collection measuring 12 mm in maximal thickness. Note the peripheral rim enhancement after contrast administration and the displacement of the overlying soft tissues. The collection extends 56 mm anteroposteriorly and 60 mm craniocaudally.

A brain CT scan was also requested, showing a scan image of a right mastoid subperiosteal abscess complicating right otomastoiditis (Fig. 5) (Table 1).

In view of this clinical condition, the patient started intravenous antibiotic therapy for 15 days with third-generation cephalosporin (Ceftriaxone 50 mg/kg/day IV) (C3G) and metronidazole (30 mg/kg/day IV), along with a short 5-day course of oral corticosteroid therapy. Drainage of the mastoid periosteal collection was also performed with a cytological and bacteriological examination with culture. Before starting antibiotic therapy, the examination failed to isolate any bacteria (Table 1).

**Figure 6.**
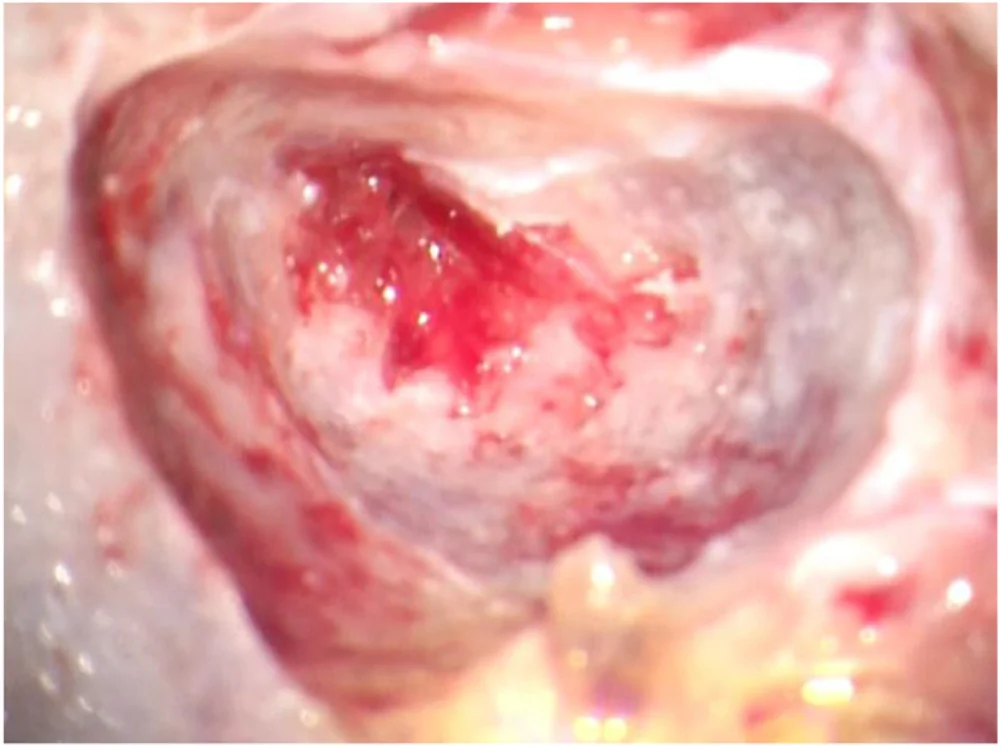
Intraoperative photograph showing the mastoidectomy drainage cavity after complete removal of inflamed mastoid air cells and drainage of purulent material. The cavity has been created to establish adequate drainage and ventilation of the infected mastoid system.

Given the lack of improvement in clinical symptoms after 15 days, a mastoidectomy for drainage was performed by ENT specialist (Fig. 6).

**Figure 7.**
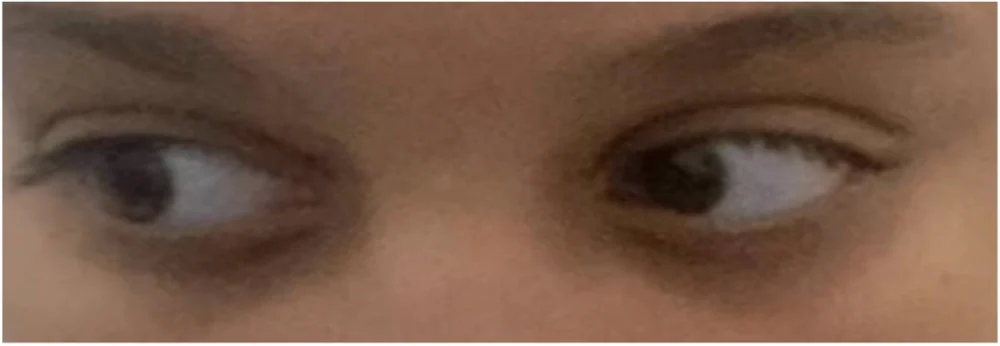
Clinical photograph obtained on postoperative day 10 demonstrating complete restoration of normal right eye mobility in abduction. Full lateral gaze is now achieved bilaterally, indicating complete recovery of abducens nerve function. This neurological recovery correlates with the resolution of retro-orbital pain observed on postoperative day 5.

Postoperatively, the patient was placed on C3G + oral metronidazole for 15 days. The course of recovery was marked by the disappearance of right retro-orbital pain on the 5th postoperative day. Normal right ocular mobility was restored on the 10th postoperative day. (Fig. 7) (Table 1).

## Discussion

Gradenigo syndrome, first described in 1904 by Italian physician Giuseppe Gradenigo, is a rare but potentially serious complication of suppurative otitis media^[^1^]^. This nosological entity, characterized by the classic triad of suppurative otorrhea, abducens nerve (VI) paralysis, and pain in the trigeminal nerve (V) distribution area, results from the spread of infection from the middle ear to the petrous apex of the temporal bone^[^2^]^.

The introduction of antibiotics has significantly changed the epidemiology of this condition, drastically reducing its incidence and mortality^[^3^]^. Nevertheless, contemporary data from recent systematic reviews show that this complication remains relevant, particularly in pediatric populations and in contexts of increasing antibiotic resistance^[^4^]^.

The current incidence of Gradenigo syndrome remains difficult to establish precisely due to its rarity. A recent pediatric systematic review by Schmit *et al* reporting 65 cases over a 20-year period suggests a stable incidence, with a predominance in school-aged children (median age: 8 years)^[^5^]^.

The petrous apex, the anteromedial portion of the petrous part of the temporal bone, is normally pneumatized in approximately 30% of the population^[^6^]^. When present, this pneumatization can facilitate the spread of infection from the mastoid cells to the petrous apex, a process known as apical petrositis.

The anatomy of this region explains the characteristic symptoms of the syndrome. The Gasser ganglion, containing the cell bodies of the trigeminal nerve, is located in Meckel’s cave, separated from the petrous apex only by a thin layer of dura mater^[^7^]^. The abducens nerve, meanwhile, passes through Dorello’s canal, located under Grüber’s petroclinoid ligament, in close proximity to the petrous apex. This anatomical proximity explains the simultaneous involvement of these two cranial nerves during inflammatory spread^[^8^]^.

The mechanism of cranial nerve involvement in Gradenigo syndrome is directly related to the anatomical relationships between the petrous apex and adjacent neurovascular structures. The infection spreads from the middle ear through the mastoid air cells to reach the petrous apex via the pneumatization pathways present in approximately 30% of the population. The Gasser (trigeminal) ganglion, which contains the cell bodies of the trigeminal nerve (cranial nerve V), lies in Meckel’s cave, separated from the petrous apex by only a thin layer of dura mater. Similarly, the abducens nerve (cranial nerve VI) passes through Dorello’s canal beneath the petroclinoid ligament of Grüber, in immediate proximity to the petrous apex. When infection reaches the petrous apex, the resulting inflammation causes edema and direct compression of these adjacent neural structures, leading to the characteristic clinical manifestations: trigeminal neuralgia-type pain (typically in the V1 and V2 distributions) and abducens nerve palsy (resulting in diplopia and impaired lateral gaze). This anatomical understanding explains why these two cranial nerves are specifically and consistently affected in this syndrome, and why the clinical presentation can occur even with relatively limited infection if anatomical proximity is particularly close^[^7,8^]^.

The mechanism of infection propagation generally follows a well-established sequence: suppurative otitis media progresses to mastoiditis, and then the infection spreads along the pneumatization pathways to the petrous apex. The local inflammation then causes edema and compression of the adjacent nerve structures. In severe cases, the condition can be complicated by osteomyelitis of the petrous apex, or even intracranial spread with abscess formation or meningitis^[^9^]^.

Modern imaging studies have provided a better understanding of this anatomical progression. MRI with gadolinium injection typically reveals enhancement of the petrous apex, adjacent meninges, and sometimes the cranial nerves themselves, reflecting the intense local inflammatory process^[^10^]^ (Table 2).

**Table 2:** Evidence-based management recommendations for gradenigo syndrome^[^5,11^]^.

Management component	Recommendations
Diagnosis	Clinical triad assessment (complete or incomplete)
	CT temporal bone with contrast (first-line)
	MRI with gadolinium (soft tissue, intracranial evaluation)
	Neurological examination (cranial nerves V, VI)
Medical therapy	First-line: Third-generation cephalosporin (Ceftriaxone 50 mg/kg/day IV, max 2 g/day)
	Add: Metronidazole 30 mg/kg/day IV for anaerobic coverage
	Duration: Minimum 21 days IV, followed by oral continuation (median 26 days)
	Consider: Short-term corticosteroids (5–7 days) to reduce neural edema
Surgical intervention	Indications: Persistent symptoms >7-10 days, abscess formation, bone destruction, neurological deterioration
	Procedures: Myringotomy (77%), ventilation tubes (70%), mastoidectomy (68%)
	Timing: Early intervention associated with better outcomes
Monitoring	Clinical assessment: Daily neurological examination
	Pain scores: Track trigeminal and retro-orbital pain
	Ocular mobility: Document abduction improvement
	Imaging follow-up: If clinically indicated
	Expected timeline: Improvement by day 11–14, complete recovery typical
Complications management	Intracranial extension: Neurosurgical consultation
	Vascular involvement: Consider anticoagulation
	Negative cultures (45% of cases): Continue empirical broad-spectrum coverage
	Treatment failure: Consider resistant organisms, alternative diagnosis

The clinical presentation of Gradenigo syndrome has evolved considerably since its initial description. A recent analysis by Schmit *et al* (2024) of 65 pediatric patients reveals that only 22% of cases present with the complete classic triad at the time of initial diagnosis^[^5^]^. This observation challenges the traditional paradigm and highlights the importance of a high degree of clinical suspicion (Table 2).

Incomplete forms, representing 74% of cases in this series, are characterized by the presence of at least two of the three cardinal signs. Abducens nerve palsy, manifested by horizontal diplopia and limited ocular abduction, remains the most frequently observed sign (74% of cases). Trigeminal pain, classically localized in the V1 and V2 areas, is reported in 77% of cases, while active otorrhea is present in only 38% of cases at the time of diagnosis^[^5^]^ (Table 2).

In children, neurological manifestations may be masked by general signs such as fever (38% of cases), altered general condition (23%), or behavioral disorders. Associated neurological complications are common (52% of cases) and include neck stiffness, photophobia, vomiting, and, in severe forms, altered consciousness^[^5^]^ (Table 2).

Serious neurological complications, although rare, remain formidable and include cavernous sinus thrombosis, intracerebral abscesses, and meningitis. The exceptional case reported by Taklalsingh *et al* (2017) describes the association of Gradenigo syndrome with Klebsiella pneumoniae bacterial meningitis, illustrating the potential severity of this condition^[^11^]^ (Table 2).

CT of the petrous bones with contrast injection is the first-line examination. It allows visualization of characteristic bone changes: opacification of the mastoid cells, trabecular lysis, and, above all, obliteration of the petrous apex, observed in 49% of cases according to the series by Schmit *et al*^[^5^]^. CT is also excellent at detecting bone complications such as osteomyelitis and allows for precise surgical planning.

MRI provides essential additional information, particularly for the evaluation of soft tissue and intracranial extension. MRI reveals an inflammatory process of the petrous apex in 84% of cases, with meningeal enhancement in 27% of cases and involvement of the internal carotid artery in 27% of cases^[^5^]^. Diffusion sequences make it possible to differentiate between inflammatory edema and purulent collection, which is crucial information for therapeutic decision-making.

The differential diagnosis must rule out other causes of oculomotor paralysis associated with cranial pain: Tolosa-Hunt syndrome, internal carotid artery aneurysm, cavernous sinus thrombosis, skull base tumors, and cavum Meckeli meningiomas. A history of recent otitis and radiological signs of petrous apex involvement point to a diagnosis of Gradenigo syndrome.

Fusobacterium necrophorum has emerged as the most frequently identified pathogen in recent series, accounting for 22% of positive isolates according to Schmit *et al*^[^5^]^. This strictly anaerobic bacterium, known for its role in Lemierre syndrome, has a particular tropism for necrotizing complications of ENT infections. Its detection requires specialized culture media and partially explains the rate of negative cultures observed in some series. Coagulase-negative staphylococci, Staphylococcus aureus, and Pseudomonas aeruginosa are the other main agents, accounting for 17%, 13%, and 13% of isolates, respectively^[^5^]^.

Antibiotic therapy is the mainstay of treatment, with intravenous administration recommended in 88% of cases according to Schmit *et al*^[^5^]^. Third-generation cephalosporins are the most commonly prescribed class of antibiotics (67% of cases in hospitals) due to their excellent spectrum of activity, good meningeal diffusion, and pediatric safety profile. Combination with a nitroimidazole (metronidazole) is recommended in 35% of cases to ensure anti-anaerobic coverage, which is particularly important given the frequency of F. necrophorum^[^11^]^ (Table 2).

The optimal duration of antibiotic treatment remains a subject of debate. The median duration of intravenous treatment reported is 21 days, followed by a median of 26 days of oral treatment^[^5^]^. This prolonged duration is explained by the need to treat potential osteomyelitis of the petrous apex and prevent recurrence. Therapeutic adjustments, necessary in 33% of cases, are mainly motivated by clinical deterioration (47%) or microbiological results (26%)^[^5^]^ (Table 2).

Surgery is required in 72% of cases, reflecting the frequent severity of this condition. Myringotomy is the most frequently performed procedure (77% of surgical cases), followed by transtympanic ventilation tube placement (70%) and mastoidectomy (68%)^[^5^]^. Direct surgical access to the petrous apex was not reported in any cases in Schmit’s pediatric series, suggesting the effectiveness of indirect drainage via mastoidectomy (Table 2).

Systemic corticosteroids are administered in 31% of cases, mainly with the aim of reducing perineural edema and improving functional recovery of the cranial nerves. However, their benefit has yet to be demonstrated in controlled studies. The management of vascular complications sometimes requires anticoagulant therapy, administered in 25% of cases according to recent series, particularly in cases of dural sinus thrombosis or carotid stenosis^[^5^]^ (Table 2).

The prognosis for Gradenigo syndrome has improved considerably with modern therapeutic advances, changing from a fatal condition to one that is generally curable with appropriate treatment. Clinical improvement is observed in 98% of cases, with complete resolution of symptoms in 73% of patients^[^6^]^. This remarkable therapeutic success rate contrasts with historical pre-antibiotic series, where mortality approached 50%. Current mortality, reduced to 2% in recent series, mainly concerns complicated forms of meningitis or brain abscesses^[^5^]^.

Recovery times vary depending on the type of procedure. Clinical improvement occurs on average 11 days after surgery and 14 days after the start of intravenous antibiotic therapy. Recovery of abducens nerve function is usually the first sign of improvement, preceding the resolution of trigeminal pain^[^8^]^.

Several prognostic factors have been identified. The presence of neurological complications at initial diagnosis (52% of cases) is associated with an increased risk of therapeutic complications and prolonged recovery times^[^10^]^. Conversely, the completeness of the initial clinical triad does not appear to significantly depend on prognosis, calling into question the old classification based on this triad (Table 2).

Despite undeniable therapeutic advances, Gradenigo syndrome continues to pose significant diagnostic and therapeutic challenges. Atypical forms, which account for the majority of contemporary cases, require an adaptation of traditional diagnostic strategies. McLaren *et al* (2020) propose a new diagnostic classification that incorporates incomplete forms and atypical presentations, which may improve early recognition of this condition^[^13^]^ (Table 2).

The key learning points for clinical practice are:

1. Maintain high index of suspicion: Incomplete forms of Gradenigo syndrome (74% of cases) may present with only two of the three classic signs. Any child with otitis media and neurological symptoms warrants immediate investigation.
2. Early neurological examination is critical: Systematic cranial nerve examination (particularly CN VI abduction and CN V sensory distribution) should be performed in all cases of complicated otitis media.
3. Imaging protocol: CT scan of temporal bone with contrast is first-line for bone detail and surgical planning, while MRI is essential for soft tissue evaluation and intracranial extension assessment.
4. Antibiotic selection rationale: Third-generation cephalosporins provide excellent central nervous system penetration and broad-spectrum coverage. The addition of anti-anaerobic agents (metronidazole) is justified by the emergence of Fusobacterium necrophorum as the leading pathogen (22% of isolates).
5. Surgical indications: Mastoidectomy is required in 72% of cases and should be considered when: (1) symptoms persist despite 7–10 days of appropriate IV antibiotics, (2) imaging shows extensive bone involvement or abscess formation, or (3) neurological deficits worsen.
6. Prolonged therapy is essential: Median treatment duration includes 21 days IV followed by oral continuation. Premature discontinuation risks recurrence and chronic osteomyelitis.
7. Monitoring for recovery: Clinical improvement typically occurs 11 days post-surgery with complete neurological recovery being the norm. Persistent deficits beyond 4 weeks warrant additional investigation for alternative diagnoses or complications.

An important limitation of this case is the failure to isolate a causative organism despite appropriate specimen collection. This finding, however, is consistent with contemporary literature where negative cultures are reported in up to 45% of Gradenigo syndrome cases. Several factors may explain this phenomenon:

- The patient’s prolonged symptom duration (1 year) suggests possible prior antibiotic exposure that may have suppressed bacterial growth;
- Fusobacterium necrophorum, now recognized as the most common pathogen (22% of positive cultures), is a fastidious anaerobe requiring specialized culture media and prolonged incubation that may not be routinely performed;

The timing of specimen collection relative to the infectious process may affect culture yield;

- The inflammatory process at the petrous apex may represent a biofilm-associated infection that is notoriously difficult to culture using standard techniques.

Despite the negative culture results, our patient’s excellent response to empirical broad-spectrum antimicrobial therapy (including anti-anaerobic coverage) validates the current evidence-based approach of initiating treatment based on clinical and radiological findings rather than delaying for microbiological confirmation. This case reinforces the importance of empirical anti-anaerobic coverage given the emerging role of F. necrophorum in Gradenigo syndrome pathogenesis.

## Conclusion

The emergence of antibiotic resistance is a growing concern. The optimization of treatment protocols must take into account changes in the bacterial spectrum and local resistance data. The use of targeted antibiotic combinations and the optimal duration of treatment require prospective multicenter studies.

Modern imaging techniques, particularly diffusion MRI and 18 F-FDG positron emission tomography, open up new diagnostic possibilities for differentiating sterile inflammation from active infection and monitoring therapeutic response^[^14^]^.

## References

[1] Gradenigo G. Über die Paralyse des Nervus abducens bei Otitis. Archiv für Ohrenheilkunde 1907;74:149–87.

[2] Matis GK de Silva DO Chrysou OI. Giuseppe Gradenigo: Much more than a syndrome! Historical vignette. Surg Neurol Int 2012;3:122. 10.4103/2152-7806.102343 PMC3512335 23226608

[3] Jensen PV Hansen MS Møller MN. The forgotten syndrome? Four cases of Gradenigo’s syndrome and a review of the literature. Strabismus 2016;24:21–27. 10.3109/09273972.2015.1130067 26979620

[4] Lutter SA Kerschner JE Chusid MJ. Gradenigo syndrome: A rare but serious complication of otitis media. Pediatr Emerg Care 2005;21:384–86. 10.1097/01.pec.0000166731.70847.d5 15942518

[5] Schmit C Keller F Gottfried T. The Diagnosis, Management, and Outcomes of Gradenigo Syndrome in Children: A Scoping Review of the Literature. Children 2024;11:1039. 10.3390/diagnostics15172193 PMC12428227 40941679

[6] Gadre AK Chole RA. The changing face of petrous apicitis-a 40-year experience. Laryngoscope 2018;128:195–201. 10.1002/lary.26571 28378370

[7] Talmor G Vakil M Tseng C. Petrous Apicitis: A Systematic Review and Case Presentation. Otol Neurotol 2022;43:753–65. 10.1097/MAO.0000000000003600 35878630

[8] Ali Z Alhantoosh S Albaqal Z. Gradenigo Syndrome: A Case Report of a Rare Complication of Otitis Media. Cureus 2024;16:e52213. 10.7759/cureus.51865 PMC10848083 38327941

[9] Ferguson MD Brent P. Gradenigo syndrome: A rare complication of otitis media. Marshall J Med 2016;2:15.

[10] Choi KY Park SK. Petrositis With Bilateral Abducens Nerve Palsies complicated by Acute Otitis Media. Clin Exp Otorhinolaryngol 2014;7:59–62. 10.3342/ceo.2014.7.1.59 PMC3932351 24587883

[11] Taklalsingh N Falcone F Ahmed I. Gradenigo’s syndrome in a patient with chronic suppurative otitis media, petrous apicitis, and meningitis. Am J Case Rep 2017;18:1039–44. 10.12659/AJCR.904648 PMC5629926 28955030

[12] Kerwan A Al-Jabir A Mathew G. Revised Surgical CAse REport (SCARE) guideline: An update for the age of Artificial Intelligence. Prem J Sci 2025;10:100079.

[13] McLaren J Cohen MS El Saleeby CM. How well do we know Gradenigo? A comprehensive literature review and proposal for novel diagnostic categories of Gradenigo’s syndrome. Int J Pediatr Otorhinolaryngol 2020;132:109942. 10.1016/j.ijporl.2020.109942 32065876

[14] Al-Juboori A Al Hail AN. Gradenigo’s syndrome and labyrinthitis: conservative versus surgical treatment. Case Rep Otolaryngol 2018;2018:6015385. 10.1155/2018/6015385 PMC6091283 30155332

